# From Cryptic Clade to Emerging Pathogen: Exploring the Evolutionary Divergence and Clinical Relevance of *Escherichia marmotae*

**DOI:** 10.3390/microorganisms14040869

**Published:** 2026-04-13

**Authors:** Pelumi Oladipo, Ayomikun Kade, Hope Onohuean, Jeffrey L. Ram

**Affiliations:** 1 Department of Physiology, School of Medicine, Wayne State University, Detroit, MI 48201, USA; 2 Department of Biochemistry, Microbiology, and Immunology, School of Medicine, Wayne State University, Detroit, MI 48201, USA; 3 School of Life Sciences, Gibbet Hill, University of Warwick, Coventry CV4 7AL, UK; ayomikun.kade@warwick.ac.uk; 4 Biomolecules, Metagenomics, Endocrine and Tropical Disease Research Group (BMETDREG), Kampala International University, Western Campus, Ishaka-Bushenyi P.O. Box 20000, Uganda; onohuean@gmail.com; 5 Biopharmaceutics Unit, Department of Pharmacology and Toxicology, School of Pharmacy, Kampala International University, Ishaka-Bushenyi P.O. Box 20000, Uganda

**Keywords:** *Escherichia coli*, *Escherichia marmotae*, cryptic clades, diagnostic tool, *Escherichia* evolution, emerging pathogen

## Abstract

The *Escherichia* genus includes both commensal and pathogenic species and is characterized by its diversity and adaptability to the mammalian gut and other environments. Among these species, *E. coli* has facilitated many scientific advances as a model organism. Recently, a new member of the *Escherichia* genus, *Escherichia marmotae*, has been described as a phylogenetically distinct clade that shows the greatest genetic divergence from *E. coli*. This review explores *E. marmotae*, its cryptic evolution, distinct characteristics, and ecological niches. *E. marmotae* has recently gained scientific prominence due to its association with animal feces, environmental occurrence, human clinical samples, and emerging as a potential pathogen. While its pathogenicity remains understudied, growing evidence from clinical, environmental, and animal sources suggests the need for heightened surveillance. This review highlights current knowledge gaps, underscores the need for improved diagnostic tools, and proposes future research directions to elucidate the clinical and ecological implications of this emerging pathogen.

## 1. Introduction

The genus *Escherichia* currently comprises six recognized species: *Escherichia coli* [1], *Escherichia fergusonii* [2], *Escherichia albertii* [3], *Escherichia ruysiae* [4], *Escherichia whittamii* [5], and *Escherichia marmotae* [6]. Among these, *E.coli* remains the most extensively studied; however, increasing attention has recently been directed toward other *Escherichia* species with emerging clinical relevance, including *E. marmotae.*

*Escherichia marmotae* was initially described as cryptic clade V, a phenotypically identical but genetically distant relative of *Escherichia coli.* However, in 2015, scientists isolated this cryptic clade from the feces of *Marmota himalaya* in Tibet, characterized its phenotypic properties, and named it *Escherichia marmotae* [6]. Since its initial isolation from Himalayan marmot feces and environmental waters [6,7], the organism has also been found in the diaphragms of wild boars [8], and the feces of cows, bank voles [9,10], clams [11], arctic fox [12] rodent [13], farm healthy hens [14], and birds [15,16,17,18]. *E. marmotae* has recently gained additional scientific prominence due to its association with human infections and genotypic virulence traits associated with *Escherichia* pathogenicity [19,20,21,22].

In 2022, *E. marmotae* was first identified in clinical samples by genome sequencing of isolates from cases of thoracic spondylodiscitis, pyelonephritis, acute sepsis of unknown origin, and postoperative sepsis [19]. The infective organisms were initially misidentified as *E. coli* using Matrix-Assisted Laser Desorption Ionization—Time of Flight Mass Spectrometry (MALDI-TOF MS), but with slightly divergent spectral profiles and low confidence scores [19]. Subsequent sequencing of their 16S rRNA amplicon unambiguously identified the organisms as *E. marmotae*, and this was later confirmed by whole-genome sequencing. Similarly, Sinha et al. (2023) identified a UTI-associated strain of *Escherichia* as *E. marmotae* [20]. The first clinical strain identification in North America was from a patient with sepsis in Detroit, MI, US [22]. The increased identification of *E. marmotae* in human infectious diseases, together with diagnostic challenges, necessitates the need for a comprehensive evaluation of this emerging pathogen. This review aims to contextualize the emergence of *E. marmotae* within the broader framework of *Escherichia* evolution. We explore the distinguishing genomic features of *E. marmotae*, its ecological reservoirs, and the mounting clinical evidence suggesting its role as a potential human pathogen. Additionally, we highlight the diagnostic challenges associated with its identification, discuss its antimicrobial resistance profiles, and provide recommendations for future research to better define its clinical and public health significance.

### 1.1. Escherichia coli and Related Species

*Escherichia coli*, a Gram-negative bacterium, is a well-known commensal resident primarily in the lower intestinal tracts of humans and warm-blooded animals, as well as water, sediment, and soil [1]. Its presence in water is often indicative of fecal contamination from warm-blooded animals [23]. Although most commensal strains of *E. coli* are relatively harmless, pathogenic strains can cause intestinal and extraintestinal diseases due to its virulence factors [24]. Its pathogenic roles span from intestinal infections, urinary tract infections (UTIs), and hospital-acquired pneumonia (HAP) to more severe conditions such as hemolytic-uremic syndrome (HUS), meningitis, and septicemia.

The *Escherichia* genus also includes other traditionally distinct species such as *Escherichia fergusonii. E. fergusonii* is genetically the most similar to *E. coli*, sharing approximately 64% DNA-DNA hybridization, which makes differentiating with 16s rRNA gene sequencing alone challenging [2,7,25]. Less similar species, such as *E. albertii* is also a member of the genus [7,26]. Traditionally, biochemical tests can be used to distinguish *E. coli* from *E. fergusonii* by its ability to ferment both lactose and sorbitol, except for certain pathogenic *E. coli* strains such as O157:H7, which do not ferment sorbitol [2,27,28].

Additionally, non-toxin producing strains of *E. coli* express the β-glucuronidase gene (*uidA*), and produce β-glucuronidase enzyme, which can be detected by a variety of different assays, including IDEXX’s Colilert, which contains two nutrient indicators, ONPG (ortho-nitrophenyl- ß-D-galactosidase) and MUG (4-methyl-umbelliferyl- ß-D- glucuronide) [29]. However, phylogenetic analysis based on housekeeping genes provides the most effective method of distinguishing *E. coli* from *E. fergusonii* and *E. albertii*, allowing for precise delineation between these closely related species [27,30].

### 1.2. Cryptic Clades Genomic Divergence and Ecological Adaptation

Despite the intensive study of *E. coli* as a model organism over a century, the discovery in the 21st century of strains that are indistinguishable phenotypically from *E. coli* but genetically divergent was particularly surprising [7]. Cryptic clades within the *Escherichia* genus were discovered through population-genetic analysis of the *uidA* gene in *E. coli* isolates from surface water, sewers, and the feces of humans, birds, raccoons, and dogs [31]. Multilocus sequence analysis based on 22 core genome genes revealed that these strains fell into five novel ‘cryptic’ lineages [7]. Evolutionary analysis using a minimum evolution tree to estimate divergence suggests that *E. albertii*, *E. fergusonii*, Clade II, and Clade V split between 38 and 75 million years ago, while *E.coli*, Clade I, Clade III, and Clade IV 19 diverged 31 million years ago [7].

**Figure 1:**
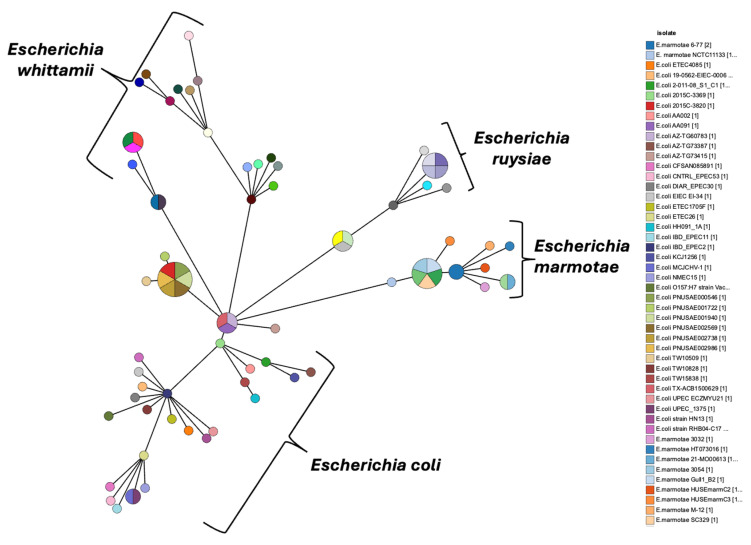
Grape tree showing the phylogenetic relationships among *Escherichia coli* and its cryptic clades.

In 2018, Cryptic Clade VI was added to the cryptic clades [32,33,34]. Recently, Mire et al. [35], reported the addition of three cryptic clades (Clade VI–Clade VIII), expanding the number to eight clades. However, the majority of available studies have focused on cryptic clades I–V, while clades VI–VIII remain mostly uncharacterized. Over the years, several cryptic clades have been renamed, such as cryptic Clade II and Clade V, which are now known as *Escherichia whittamii* and *Escherichia marmotae* respectively, while *Escherichia ruysiae* sp. encompasses the closely related *Escherichia* Cryptic Clades III and IV (Figure 1, Table S1). Pairwise comparison of the full genomes of isolates of *E. marmotae* to representative sequences of *E. coli* indicated that these genomes differed on average by 9.4% over all genes [36]. Genetic distances from *E. coli* to various cryptic clades estimated from single-nucleotide polymorphisms (SNPs) of core genome genes averaged 3.2% for Clade I, 7.5% for Clade II, 6.7% for Clade III, 6.5% for Clade IV, and 8.1% for Clade V, emphasizing the greater divergence of Clade V (*E. marmotae*) from *E. coli* in comparison to the other cryptic clades [37].

The cryptic clades inhabit ecological niches different from those of *E. coli.* Clades II–V are relatively abundant and well-suited to non-intestinal environments, such as freshwater beaches and surface water [33]; whereas, Cryptic Clade I favors the intestinal environments where they appear to provide functions that enhance environmental survival, such as diol utilization and lysozyme production [38,39]. Cryptic clades have also been identified in other animal hosts, particularly birds [16,40]. The cryptic clades have been shown to have the potential to cause extraintestinal infections through the expression of distinct virulence factors. Interestingly, Ingle et al. [41] revealed that Cryptic Clades III, IV, and V form biofilms better than the *E. coli*, *E. albertii*, and *E. fergusonii* strains. They can replicate at temperatures as low as 5 °C, unlike *E. coli* and *E. fergusonii*, which usually require at least 11 °C. While a mouse model of extraintestinal infection shows they are non-virulent, their ability to survive in harsh environments, coupled with their robust biofilm formation, raises concerns about their potential as opportunistic pathogens [41].

## 2. Molecular Detection and Pathogenic Potential of Cryptic Clades

Historically, identifying and differentiating non-*E. coli* from *E. coli* strains [7] relied on sequencing specific genes, which is time-consuming and labor-intensive. However, in 2011, Clermont et al. developed a PCR-based technique to distinguish *E. coli* from various cryptic clades of *Escherichia* [40]. The approach involved allele-specific PCR reactions targeting the *chuA*, outer membrane heme, and *aes* (acetyl esterase B) genes with clade-specific primers, resulting in PCR products of distinct sizes, unique to each clade. The method proved effective even when traditional tests failed to differentiate them. This coincidentally identified the first suspected instances of cryptic clades (Clades III and V) associated with human infections, since these isolates were from a large collection of *Escherichia* isolates from septicemic patients in France [42].

A study by Vignaroli et al. [39] identified 20 cryptic clades among 138 *E. coli* strains isolated from marine sediments, using the *aes* and *chuA* allele-specific primers developed by Clermont et al. [40] to investigate the pathogenic potential of the cryptic clades. The study assessed the adhesion and invasion ability of the cryptic clades using Caco-2 and Int-407 mammalian intestinal cell lines. Among the cryptic clades, Cryptic Clade V had the highest adhesion index, comparable to the positive control *E. coli* ATCC35150 [39]. However, none of the cryptic clades could invade intestinal cell lines. This lack of invasive ability of Cryptic Clade V on epithelial cells was also observed in a study by Zhurilov et al. [9]. In contrast, in 2019 Liu et al. [43] demonstrated that *E. marmotae* possesses some invasive capacity, albeit with a lower invasion index than *Shigella*, a known invasive pathogen. These results indicate that various *E. marmotae* strains may differ in their invasive capability and thereby in their potential to act as invasive pathogens.

Vital et al. [44] investigated the gene expression profiles of *Escherichia* Cryptic clades under different environmental conditions. *Escherichia* Cryptic clades exhibit high expression levels of stress defense genes under starvation conditions, upregulating genes associated with acid stress defense, desiccation stress defense, and toxic compound stress defense, thus enhancing the capacity for survival [44]. These adaptations ensure survival and resilience in adverse conditions. Cryptic clades isolated from marine sediments [45] were studied for biofilm formation, resistance to hydrogen peroxide, and expression of the “red, dry, and rough” (rdar) morphotype, a phenotype characterized by the production of extracellular matrix composed of curli fimbriae and cellulose [46]. Out of 28 cryptic clade isolates tested, 79% were weak or non-producers of biofilm at 37 °C and lacked the surrounding extracellular matrix. However, growth at 24 °C led to significant biofilm formation and expression of the rdar morphotype [45]. The co-expression of curli and cellulose plays an important role in biofilm formation, cell aggregation, and cell adhesion [45,47]. Future work to characterize how genomic variations among the cryptic clades cause phenotypic changes are potentially important for determining their pathogenicity.

Although initial studies suggested that *E. whittamii*, *E. ruysiae*, and *E. marmotae* were not significant public health risks [38], recent reports of *E. marmotae,* associated with human infections [19,20] indicate a potential pathogenic role for *E. marmotae*. Further investigation is necessary to fully understand its pathogenic potential.

## 3. Cryptic Clades as Indicators of Fecal Contamination in Water Quality Monitoring

Fecal contamination of recreational waters is a significant source of pathogens that cause waterborne diseases. *E. coli* has been the gold standard indicator of fecal contamination [48]. Conventional testing methods enumerate *E. coli* by detecting the expression of the *uidA* gene, which encodes beta-D-glucuronidase enzyme [29,30]. Detection of beta-glucuronidase-positive colonies and cultures is recommended by the US Environmental Protection Agency (USEPA) for monitoring fecal pollution in water (USEPA, 2002). However, other species within the genus *Escherichia* also possess this gene, potentially leading to confounding interpretations of water quality, as environmental bacteria do not indicate the presence of harmful fecal material.

Environmentally adapted “naturalized *E. coli*” may contribute to an increased number of *E. coli* counts and lead to false alarms of fecal pollution. Several studies have suggested detecting “cryptic clades” in the current *E. coli*-based water quality monitoring methods might overestimate the health risk of faecal contamination [7,40,49]. To address this issue, Mire et al. [35], developed a specific PCR assay targeting a gene called *ecc* (*Escherichia* Cryptic clades), which is specific to only cryptic clades as confirmed by sequencing their whole genomes. Deng et al. [50] also proposed that the *ycjM gene*, a putative glycosyltransferase gene, is a distinctive genetic marker to differentiate enteric *E. coli* from naturalized *E. coli*. The study hypothesised that *ycjM* is present in host intestinal tracts and absent in environmental *E. coli* and non-*E. coli* bacteria [50]. Genetic techniques have been developed to discriminate enteric–fecal indicator bacteria from naturalized *E. coli* by the presence of enteric-associated genes for gut colonization and several prophage genes [38,51].

Ecological analysis has shown that members of Clade I are often isolated from human and animal feces [41,52], indicating their potential as fecal indicator bacteria [35]. In contrast, isolates of clades II through V are mostly collected from water or soil samples [7,33,39,40]. *E. marmotae* and *E. ruysiae* have been isolated from environmental, animal, and human sources, and their genotypic characteristics are similar to those of Cryptic Clade I, presenting a compelling case for their inclusion as fecal indicators alongside Cryptic Clade I [19,53]. The variation in these clades across environments, coupled with the inconsistent presence of the *ycjM* gene among strains, underscore that they may be identified as indicators of fecal pollution.

## 4. Cryptic Clades

### 4.1. Cryptic Clade I

Cryptic Clade I exhibits the characteristic virulence traits of *E. coli*, including their potential to cause human infections [40,41,52]. Thus, *E. coli* and *Escherichia* Clade I are now designated as *E. coli* sensu lato, and the classic *E. coli* (designated phylogroups A–G) are referred to as *E. coli* sensu stricto, which are genetically closer to *E. coli* than other clades [54]. Cryptic Clade I are predominantly enteric, commonly found in animal gastrointestinal tracts, and less frequently in external environments [7,38,41]. These strains have been implicated in human infections, including those isolated from children with diarrhea.

Significant findings indicate that clade I strains capable of producing ETEC enterotoxins, as well as STEC strains, have been isolated from human patients [33,52] and from animals [32,33,55]. Distinguishing clade I from *E. coli* is important because Clade I strains harbour virulence factors that cause human diarrhea. Okuno et al. [56] developed a PCR-based method to distinguish Clade I from *E. coli* to help avoid false identification of cryptic clades. The method relies specifically on a gene of unknown function, 1368 bp in length, that contains two secreted effector protein pipB2 domains, which are conserved in clade I strains and absent in other members of the Enterobacteriaceae [56].

### 4.2. Escherichia whittamii

Cryptic clade II was named *Escherichia whittamii* in honor of the American microbiologist Thomas S. Whittam [5]. Whittam made significant contributions to microbiology, particularly in the collection and characterization of *E. coli* strains and the identification of the cryptic clades. Although *E. whittamii* shares its genus with the well-known *E. coli*, it remains under-researched and is considered rare [40]. It has been isolated from diverse biological sources, including bird feces [7], chicken gut [5,14], and human fecal samples [40], indicating its widespread presence. However, the ecological niche and characteristics of *E. whittamii* need further exploration.

Recent studies by Shen et al. [57,58] investigated the genetic diversity and potential adaptation of strains of *E. whittamii* isolated from intertidal sediments. Through comprehensive genomic analysis, they revealed ecological differentiation within this cryptic clade. Delta bitscore metrics, a method for quantifying sequence alignments, were used to assess evolutionary divergence within *E. whittamii* [59]. The study identified unique genetic signatures indicative of *Escherichia whittamii’s* adaptation to distinct ecological niches.

### 4.3. Escherichia ruysiae

*Escherichia ruysiae*, formerly classified within cryptic clades III and IV, has been predominantly isolated from environmental samples [7]. However, its detection in a range of animal and human-associated sources indicates a wider host range than previously recognized [60]. *E. ruysiae* was first isolated from a fecal sample of an international traveler with diarrhea [4], demonstrating its ability to colonize the human gastrointestinal tract. More recently, a clinical isolate was recovered from a five-year-old female patient hospitalized with gastroenteritis and bloody diarrhea, further suggesting its potential relevance in human disease [61].

Beyond human-associated cases, *E. ruysiae* has been detected in multiple animal reservoirs. Isolates have been recovered from a Lohmann Brown layer hen (*Gallus gallus* domesticus) on a farm in England [14] and wild crow in Japan [18]. Its presence in the fecal microbiome of a healthy domestic dog in the United States highlights its ability to colonize companion animals and supports its broad geographic distribution [60]. Collectively, these findings suggest that *E. ruysiae* is geographically widespread, with reports spanning North America, Europe, Asia, and Africa [4,8,18,60,61,62].

Despite its detection across diverse hosts and environments, the ecological distribution, transmission pathways, and potential role of *E. ruysiae* in human and animal health remain poorly characterized. Genomic analyses have identified several virulence- associated genes, such as those involved in siderophore function (*chuX*, *entS*, *fepABD*), fimbriae (*fimBCDGI*), heat-stable enterotoxins (*astA*), adhesion and biofilm formation genes (*csgABCDEFG* and *agn43*), chemotaxis regulatory protein (*CheRBYZ*), *ompT* gene for outer membrane protease, flagellar protein (*flgABCDE*, *fliABC*, *flhABC*, and *motAB),* fitness *(iutA* and *kpsMII),* type II secretion systems (*gspGHI*), and capsular polysaccharide biosynthesis (*kpsD*), none of which are strictly associated with a defined *E.coli* pathotype. Some of them can be found in nonpathogenic *E.coli* [8,18,61,62,63].

A smaller subset of *E. ruysiae* isolates harbor virulence determinants more commonly associated with pathogenic *E. coli*, including dispersin (*aap/aspU*), the aat secretion system (*aatABCP*), enterotoxin gene *senB*, colicin Ib (*cib*), plasmid R100 complement resistance gene (*traJT*), all of which may contribute to diarrheal infections [18,61,62]. Additionally, the presence of the *sat* gene typically associated with uropathogenic *E. coli* (UPEC) and enteroaggregative *E. coli* (EAEC), further suggests the potential for pathogenic behavior [63]. However, no consistent association between *E. ruysiae* and specific clinical syndromes, including diarrheal disease, has been established, complicating efforts to define its pathogenic capabilities [4,11,62].

*Escherichia ruysiae* harbors a range of antimicrobial resistance genes (ARGs), including extended-spectrum β-lactamases (ESBLs) such as *bla_CTX-M-15_*, *bla_CTX-M-14_*, and *bla_AmpC_,* variants, indicating resistance to β-lactam antibiotics [4,60,61,62]. Additional resistance determinants include genes associated with aminoglycosides (*aadA*, *aph*) [4,60,62], fluoroquinolones (*qnrS1*) [62], trimethoprim (*dfrA*) [62], sulfonamides (*sul2*) [4,62], and tetracycline (*tet(A)* [62]), as well as multidrug efflux systems (*emr*, *edt*) [61].

Accurate identification of *E. ruysiae* presents significant challenges in clinical laboratory settings. Conventional biochemical identification methods and automated systems such as VITEK2 and MALDI-TOF mass spectrometry frequently misidentify *E. ruysiae* as *E. coli* due to highly similar phenotypic characteristics [4,18,60,61,62]. At present, *E. ruysiae* is not considered a human pathogen, and its role as a zoonotic pathogen in human populations remains unclear. Further studies and enhanced surveillance are needed to understand its ecological roles and resistance mechanisms and potential contribution to human disease.

### 4.4. Escherichia marmotae

*E. marmotae* has been found in various environments, inhabiting the diaphragms of wild boars, the rectal feces of cows [10], bank voles [9], farm healthy hens [5], clams [11], birds [17,18], and wild boars [8]. The prevalence of human infection caused by *E. marmotae* remains unclear [18,19,20,22].

This broad host range suggests that *E. marmotae* may represent an emerging pathogen that has been overlooked amidst the many invasive *E. coli* strains processed in clinical microbiology laboratories. Historically, MALDI-TOF MS could not reliably distinguish *E. marmotae* from *E. coli* [18,19,21,64,65]; however, detection has recently improved with the identification of a discriminatory spectral peak at 7260–7268 m/z [22]. Numerous human infections associated with *E. marmotae* have now been documented (Table 1), with most cases initially misidentified as *E. coli*.

#### 4.4.1. Phenotypic Characterization and Physiology of *Escherichia marmotae*

*E. marmotae* is a Gram-negative, facultatively anaerobic rod [6]. Phenotypically, it closely resembles *Escherichia coli*, which contributes to its frequent misidentification in routine clinical microbiology laboratories. *E. marmotae* exhibits an *Escherichia*-like biochemical profile; catalase-positive, oxidase-negative, hydrogen sulfide (H_2_S) negative, and urease-negative [6,9,21]. However, strain-level variability has been reported in several biochemical reactions, including indole production, β-galactosidase (ONPG) activity, lactose fermentation, ornithine decarboxylase activity, and carbohydrate utilization profiles. This variability likely contributes to its frequent misidentification as atypical or inactive *E. coli.*

**Figure 2:**
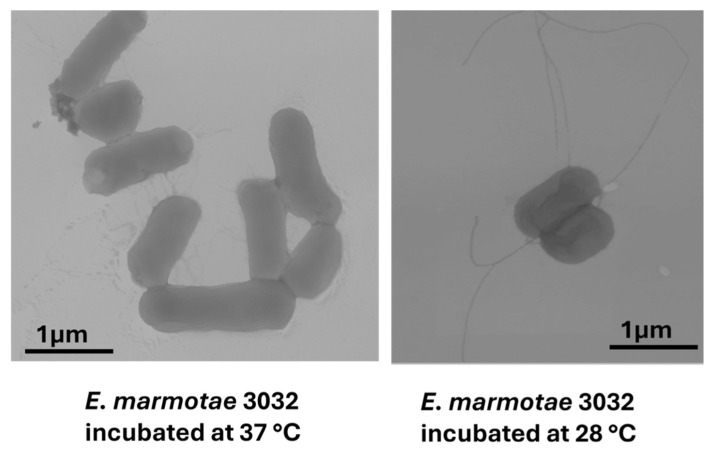
Transmission electron micrographs of *E. marmotae* 3032. The panels illustrate temperature-dependent flagellar expression, with flagella present after incubation at 28 °C (**right panel**) and absent/reduced at 37 °C (**left panel**). Image adapted from Oladipo et al. 2025 [36].

Among the *Escherichia* cryptic clades, *E. marmotae* is the most studied, but many areas still need investigation. Contradictory reports about the motility of *E. marmotae* have been published, with some identifying it as motile at human body temperature [9] and others as non-motile [6,13,22,64]. A recent study showed that *E. marmotae* is motile and possesses flagella at 28 °C but not at 37 °C (Figure 2) [36]. Further investigation is needed as motility could significantly impact the infection process. Temperature-dependent changes in motility have been observed in *E. marmotae* and require further investigation, especially regarding the genes that mediate these behaviors [36].

Similarly, biofilm formation in *E. marmotae* appears to be influenced by temperature [36]. Studies have shown that they produce more robust biofilms at lower temperatures [36,41], than at 37 °C, consistent with increased expression of extracellular matrix components, such as curli fimbriae and cellulose. At host temperature, biofilm formation is reduced, and extracellular matrix production may be diminished despite the presence of underlying genetic determinants, as reported for cryptic *Escherichia* clades [45]. This indicates that environmental cues play a significant role in regulating surface-associated phenotypes.

#### 4.4.2. Genomic Features, Virulence, and Antibiotic Resistance

The genome size of *E*. *marmotae* typically ranges from 4.2 to 5.2 Mbp, making it slightly smaller on average than *E.coli* [6,10,13,36,53,64]. As noted above in Section 1.2, the average nucleotide identity (ANI) based on whole-genome sequences shows about 9% similarity between *E. marmotae* and *E. coli* and other Cryptic clades, with intra-species similarity >98% [36,66]. Based on 16S rRNA gene sequences, *E. marmotae* exhibits >99.9% identity to other *E. marmotae* strains and approximately 99.2% similarity to *E. coli* [6,9,13,20]. Comparative genomic analyses further show that *E. marmotae* strains share more than 3000 core genes and a large accessory genome [19,36].

Various studies have identified numerous virulence genes within *E. marmotae* in environmental and clinical strains [6,17,19,20,21,22,36,64]. These include the enterotoxin encoding gene *astA*, iron acquisition systems (*entA/B/C/D/E/F/S*), heme uptake (*fecA/B/C/DE*) and metabolism (*chuS/T/U/V/W/X*), and genes involved in the production of curli proteins (*csgB/D/E/F/G*) and type 1 fimbriae (*fimA/B/C/D/E/F/G/H/I*), adherence, regulatory factors (*gadX, H-NS*, *marA*), bacterial secretion systems (*gspC/D/E/F/G/H/I/J/K/L/M*), and invasion factors (*lbeA/B/C*) [6,8,9,19,20]. These genetic components aid bacterial survival during invasive infections by promoting cellular adhesion, biofilm formation, and increased virulence. Most strains of *E. marmotae* possess outer membrane protein A, which has been associated with pathogenic properties that enable bacteria to breach the blood–brain barrier and evade host defense mechanisms, as observed in Neonatal meningitis *E. coli* (NMEC) [67].

Experimental studies suggest that virulence in *E. marmotae* may be strain-dependent. A recent study by Zhurilov et al. [9] examined a single *E. marmotae* strain (M-12) isolated from the lungs of a wild bank vole. When administered to mice, M-12 did not exhibit measurable virulence, consistent with earlier observations by Ingle et al. [41]. Despite this, the strain showed strong adhesion to HEp-2 cells and red blood cells, likely due to curli and fimbrial genes. Although biofilm genes were present in the genome, M-12 did not form biofilms at 37 °C [9], consistent with reports that *E. marmotae* exhibits enhanced biofilm formation at lower temperatures [36,41].

Plasmid-associated virulence has also been reported. Among the strains of *E. marmotae*, a unique isolate from the feces of the Himalayan marmot (*Marmota himalayana*) has been identified with a type III secretion system (T3SS) and other virulence genes found within the pEM148 plasmid. This plasmid of *E. marmotae* HT073016 [6] exhibits a genetic similarity ranging from 45% to 94% with a large virulence plasmid pCP301 (pINV) of *Shigella flexneri*, with almost the entire gene cluster but lacking the *virF* gene, a master activator needed for expression of the invasive phenotype [68]. Cell invasion experiments also showed that this strain is less invasive than *Shigella*, an invasive pathogen [43].

Genetic variability has been observed for mobile genetic elements (MGE), plasmids, and prophages, supporting the frequent gene gain and loss across the species [64]. Plasmid analyses reveal the presence of multiple incompatibility (Inc) groups, including IncF, IncI, and IncN, many of which are associated with conjugative transfer. Notably, *E. marmotae* has been shown to acquire clinically relevant resistance plasmids, such as IncL plasmids carrying *bla*_OXA-48_ [65] as well as AmpC- and ESBL-associated genes shared with co-existing *E. coli* populations [17]. Antimicrobial resistance genes are frequently linked to transposable elements, highlighting the role of MGEs in facilitating gene acquisition and dissemination. Collectively, these findings indicate that *E. marmotae* harbors a dynamic mobilome that supports adaptation across diverse ecological niches and contributes to the spread of antimicrobial resistance.

Reports of antibiotic resistance in *E. marmotae* have increased in recent years [11,17,21]. A multidrug-resistant (MDR) *E. marmotae* strain was reported from a 64-year-old man hospitalized with febrile neutropenia—the only documented MDR case in the Czech Republic [21]. Resistance to several antibiotics has been reported in both environmental and clinical isolates, including ampicillin [9,21], amoxiclav [9], tobramycin [9], tetracycline [17,36], ceftazidime [21], cefotaxime [21], cefazolin [36], erythromycin [13,36], and streptomycin [17]. Thus, *E. marmotae* has the capacity to acquire antimicrobial resistance, possibly through horizontal gene transfer and plasmid-mediated mechanisms. The apparent underreporting of resistant *E. marmotae* may be partly attributable to its frequent misidentification as *Escherichia coli* in routine clinical diagnostics, which could obscure its true contribution to antimicrobial resistance surveillance.

An *E. marmotae* strain isolated from clams was identified as an ESBL (Extended-Spectrum Beta-Lactamase) producer, showed phenotypic resistance to cefotaxime, and harbored the *blaCTXM-1* gene [11]. Strains of *E. marmotae* isolated from a patient with a urinary tract infection contained *blaKPC*, *blaCTX-M*, and *blaTEM-1b* [20], highlighting the clinical relevance of *E. marmotae* in infections involving antimicrobial resistance.

*E. marmotae* exhibits variations in O antigens, and all *E. marmotae* have been shown to contain the *fliC*-H56 flagella antigen in both human and environmental strains [18,19]. *E. marmotae* may be truly pathogenic and able to cause severe infections. *E. marmotae* isolated from cow rumen was capable of potential biohydrogen production from sugarcane molasses under dark fermentation conditions [69]. *E. marmotae* has been reported to produce uricase by forming halo zones on uric acid agar plates while investigating potential uricase-producing strains from canal water near a dairy [70]. Uricase is an enzyme that facilitates the oxidation of uric acid into allantoin in the presence of oxygen. The enzyme has been used as a treatment to reduce the buildup of toxic urate, thereby serving as a therapeutic approach for managing gout and neuropathy [71].

Evidence points towards *E. marmotae* sharing similar pathogenic traits to extraintestinal pathogenic *E. coli* (ExPEC) and Enteroaggregative *E. coli* (EAEC). These traits are partly mediated by gene *aaic* and other gene components of the type VI secretion system [18]. ExPEC includes strains such as avian pathogenic *E. coli* (APEC), which share significant genetic similarities with human pathogenic strains, suggesting a potential zoonotic transfer of virulence and resistance genes [72].

The prevalence of illnesses caused by *E. marmotae* remains largely unknown, as does its role as a potential commensal organism in humans and animals. Given its shared genetic and phenotypic characteristics with pathogenic *E. coli* and the lack of a robust clinical test for *E. marmotae*, it seems likely that its occurrence is underreported. Another area that has hardly been explored is the influence of environmental factors on the expression of genes involved in virulence. These insights into the genomic and phenotypic variability of *E. marmotae* emphasize the necessity for research to understand better its clinical prevalence, pathogenic potential, and ecological roles.

## 5. Future Directions for Research Engagement

The classification of *E. marmotae* as either a human or an opportunistic pathogen remains an open question that requires further clarification. While some evidence suggests that *E. marmotae* has pathogenic potential, its role as a commensal organism in various environments and hosts complicates its categorization. Future research should focus on identifying the specific conditions under which *E. marmotae* transitions from a commensal to a pathogenic role. This includes investigating host-specific factors that may predispose individuals to infection, as well as environmental triggers that could influence its virulence. The exploration of genetic, ecological, and environmental factors influencing *E. marmotae*’s pathogenic potential will be critical in refining its classification and understanding its true clinical significance.

Improved clinical diagnostic methods for identifying the various cryptic clades are needed. MALDI-TOF MS has the potential to provide robust routine identification; however, several MALDI databases, including VITEK MS Prime by bioMérieux, lack spectra for *E. marmotae.* Bruker MALDI-TOF-MS includes *E. marmotae* in its Research Use Only (RUO) database, but independent documentation of its reliability is lacking. Updating these databases to include spectra for *E. marmotae*, *E. ruysiae*, and *E. whittamii* is critical to properly diagnosing *Escherichia* infections. Extensive research should focus on identifying unique peaks that distinguish these species from closely related pathogens, enhancing the diagnostic capabilities of MALDI-TOF MS. This technique has been used successfully to differentiate closely related bacterial species [73,74,75] and would significantly enhance the diagnostic capability of MALDI-TOF MS and support the early detection of infections caused by these organisms.

Comprehensive genomic studies are essential to uncover functional and genetic distinctions among *E. coli*, *E. marmotae,* and other *Escherichia* species. Understanding these differences will be crucial to assessing the pathogenic potential of the various *Escherichia* species, especially the roles of *E. marmotae* and the other formerly cryptic clades as potential zoonotic pathogens. Further research into how temperature-dependent conditions affect motility, biofilm formation, and virulence gene expression in *E. marmotae* will provide deeper insights into its environmental adaptability and potential pathogenic behavior in warm-blooded animals.

## 6. Conclusions

The study of *E. marmotae* has revealed its potential role as a human pathogen, harboring virulence factors, and being associated with various infections. Including these species in water quality monitoring will enhance the detection of fecal contamination and potential public health risks. Given its 91% genetic similarity to *E. coli*, there is a need for improved diagnostics. Investigating these genes, particularly those related to motility and temperature-dependent behavior, will provide insights into the adaptations and pathogenic mechanisms of these formerly “cryptic clades” of *Escherichia*.

## Figures and Tables

**Table 1:** Clinical cases of *E. marmotae* associated with human infections.

Source of Infection	Diagnostic Methods	Treatment	Country	References
80-year-old male with acute mylgenous leukaemia and pancytopenia diagnosed with thoracic spondylodiscitis	MALDI-TOF misidentified it as *E. coli* 16S rRNA sequencing	Expanded-spectrum cephalosporin followed by a course of oral ciprofloxacin.	Norway	[19]
Pyelonephritis	MALDI-TOF misidentified it as *E. coli* 16S rRNA sequencing	Not provided	Norway	[19]
Healthy male admitted acute sepsis of unknown origin	MALDI-TOF misidentified it as *E. coli* 16S rRNA sequencing	Expanded-spectrum cephalosporin	Norway	[19]
66-year-old male with a history of gallstone disease developed acute postoperative sepsis with multiple organ failure.	MALDI-TOF misidentified it as *E. coli* 16S rRNA sequencing	Intravenous piperacillin–tazobactam followed by oral trimethoprim–sulfamethoxazole	Norway	[19]
Patient had Urinary tract infections	MALDI-TOF 16S rRNA sequencing	Not provided	Australia	[20]
64-year-old man undergoing chemotherapy for urinary tract cancer was hospitalized with febrile neutropenia.	MALDI-TOF misidentified it as *E. coli* 16S rRNA sequencing	Patient died	Czech Republic	[21]
Sepsis	MALDI-TOF misidentified it as *E. coli* Confirmed with Whole-Genome Sequencing (WGS).	Not provided	U.S.	[22]

## Data Availability

No new data were created or analyzed in this study. Data sharing is not applicable to this article.

## Supplementary Materials

The following supporting information can be downloaded at: https://www.mdpi.com/article/10.3390/microorganisms14040869/s1, Table S1: Strains of Escherichia coli and cryptic clades used for phylogenetic tree.

## Abbreviations

The following abbreviations are used in this manuscript:

ONPG	Ortho-nitrophenyl-ß-D-galactosidase
UTI	Urinary tract infections
MUG	4-methyl-umbelliferyl-ß-D-glucuronide
HUS	Hemolytic-uremic syndrome
HAP	Hospital-acquired pneumonia
MALDI-TOF MS	Matrix-Assisted Laser Desorption Ionization—Time of Flight Mass Spectrometry
ESBLs	Extended-Spectrum β-lactamases
T3SS	Type III secretion system
RUO	Research use only
ANI	Average Nucleotide Identity
ExPEC	Extraintestinal Pathogenic *E. coli*
EAEC	Enteroaggregative *E. coli*
APEC	Avian Pathogenic *E. coli*
USEPA	US Environmental Protection Agency
